# Air‐Stable Copper‐Based P2‐Na_7/9_Cu_2/9_Fe_1/9_Mn_2/3_O_2_ as a New Positive Electrode Material for Sodium‐Ion Batteries

**DOI:** 10.1002/advs.201500031

**Published:** 2015-05-04

**Authors:** Yunming Li, Zhenzhong Yang, Shuyin Xu, Linqin Mu, Lin Gu, Yong‐Sheng Hu, Hong Li, Liquan Chen

**Affiliations:** ^1^ Key Laboratory for Renewable EnergyBeijing Key Laboratory for New Energy Materials and DevicesBeijing National Laboratory for Condensed Matter PhysicsInstitute of PhysicsChinese Academy of SciencesBeijing100190China; ^2^ Laboratory for Advanced Materials and Electron MicroscopyInstitute of PhysicsChinese Academy of SciencesBeijing100190China

**Keywords:** copper redox couple, Na_7/9_Cu_2/9_Fe_1/9_Mn_2/3_O_2_, layered oxides, sodium‐ion batteries, grid storage

## Abstract

**An air‐stable copper‐based P2‐Na_7/9_Cu_2/9_Fe_1/9_Mn_2/3_O_2_** is designed and synthesized by a simple solid‐state method and investigated as a positive electrode material for sodium‐ion batteries. The attractive long cycling stability is demonstrated by the capacity retention of 85% after 150 cycles at 1 C rate without phase transformation. The reversible Cu^2+^/Cu^3+^ redox couple in P2 phase oxides is proved for the first time.

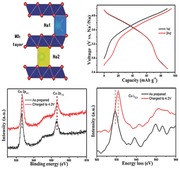

Lithium‐ion batteries have been successfully used as a power source for portable electronics during the last two decades, and they are now also a promising alternative source for electrical vehicles applications. However, the production cost of lithium and its non‐uniform geographic distribution may limit their further application in the large‐scale energy storage.^1^, ^2^, ^3^ Therefore, the development of inexpensive large‐scale energy storage with long cycle life and high energy conversion efficiency is very urgent for integration of renewable energy sources into the electrical power grid. Recently, sodium‐ion batteries have attracted worldwide attention due to unlimited resources and low cost of sodium, and they will be a viable alternative to lithium‐ion batteries in the near future.^4^, ^5^, ^6^, ^7^, ^8^

Up to now, several negative electrode materials of sodium‐ion batteries have been proposed and show good performance. They mainly include amorphous carbon materials, alloys, Ti‐based oxides, and organic compounds.^9^, ^10^, ^11^, ^12^, ^13^, ^14^, ^15^, ^16^, ^17^, ^18^ Recently, Hu et al. reported an amorphous monodispersed hard carbon spherule carbonized at 1600 °C with a high capacity (310 mAh g^−1^) and excellent cycling performance.[[qv: 10b]] Alloy‐negative electrode materials of Sb/C,^11^ Sn/C,^12^ and SnSb/C^13^ exhibit a high capacity of 610, 295, and 540 mAh g^−1^, respectively. Ti‐based oxides mainly include Na_2_Ti_3_O_7_,^14^ Li_4_Ti_5_O_12_,^15^, ^16^ Na_0.66_[Li_0.22_Ti_0.78_]O_2_,^17^ MgTi_2_O_5_,^18^ and so on.

Searching for suitable positive electrode materials is an important step to implement the application of sodium‐ion batteries. Over the past decade, numerous materials have been studied as possible positive electrode materials for sodium‐ion batteries; they mainly include layered oxides, tunnel‐type oxides, phosphates, and sulfates.^23^, ^24^ Although polyanionic compounds show the structure stability during the Na extraction/insertion process, the kinetics for Na insertion in most of them is sluggish. As a kind of tunnel‐type oxide, orthorhombic Na_0.44_MnO_2_ (Pbam) with a large S‐shaped tunnel structure was investigated by Doeff et al.^25^ as a positive electrode of sodium‐ion batteries for the first time, and then Cao et al.^26^ reported good rate capability and excellent cycle life of Na_0.44_MnO_2_ nanowires. However, only ≈0.22 Na can reversibly be cycled for the practical applications in a full cell. One of the most promising families of positive electrode materials is layered oxides with a general formula of Na*_x_*MO_2_ (M = Ni, Co, Mn, Fe, Cr, etc.) because of their high capacity and easy preparation.^27^, ^28^, ^29^, ^30^, ^31^, ^32^, ^33^, ^34^, ^35^ Layered oxides can mainly be classified into P2 and O3 according to the oxygen stacking sequence and coordination environment of the alkali ions.^36^ Although O3‐type compounds have higher reversible capacity, their cycling stability is not good and they are not stable in air.^27^, ^28^, ^29^, ^30^, ^31^, ^32^, ^33^, ^34^, ^35^, ^36^, ^37^, ^38^, ^39^, ^40^, ^41^, ^42^, ^43^, ^44^ In contrast, the P2 phase materials deliver better cycle performance and rate capability because the larger trigonal prismatic site occupied by Na^+^ ion is beneficial for the transport of Na^+^ ion.^45^, ^46^, ^47^, ^48^, ^49^, ^50^, ^51^ P2‐Na_2/3_Fe_1/2_Mn_1/2_O_2_ delivers the highest charge/discharge capacity of 190 mAh g^−1^ in a sodium half‐cell within the voltage range of 1.5–4.3V, but its cycle performance is insufficient and polarization is relatively large.^45^ Furthermore, most of P2 materials are hygroscopic, one exception example is P2‐Na_2/3_Ni_1/3_Mn_2/3_O_2_ that is very stable against water.[[qv: 10b,46]] As a matter of fact, only layered oxides containing Ni or Co transition metal show promising Na storage performance in terms of high storage capacity and long cycling stability.^38^, ^39^, ^40^, ^41^, ^42^, ^46^, ^47^, ^48^, ^49^, ^50^, ^51^ However, their large‐scale application of Ni and Co in lithium‐ion batteries for electric vehicles would further increase their price; thus, they are unfavorable for the sodium‐ion battery system.

In contrast, copper is harmless and the cost of copper oxide is only half of that of nickel oxide. Recently, Hu and co‐workers^52^, ^53^ reported the electrochemistry of P2 and O3 phase oxides with transition metal element of Cu, realizing the reversible Cu^2+^/Cu^3+^ redox couple for the first time. For common P2 phase oxides, the sodium content is the typical 2/3, and phase transformation occurs when the sodium content is less than 1/3.^46^ The capacity of P2‐oxides is limited by the sodium content; thus, increasing the sodium content is crucial for improving the capacity and suppressing the phase transformation. Here, we aim to design an air‐stable P2‐phase oxide with non‐toxic elements and a high sodium content. First, Ni was entirely replaced by Cu in the Na_2/3_Ni_1/3_Mn_2/3_O_2_ to obtain an air‐stable P2‐Na_2/3_Cu_1/3_Mn_2/3_O_2_. Next, we intentionally introduced a cheaper element into the structure of P2‐Na_2/3_Cu_1/3_Mn_2/3_O_2_ to partially replace Cu to improve the sodium content and the structural stability. Finally, we succeeded in the synthesis of an air‐stable P2‐Na_7/9_Cu_2/9_Fe_1/9_Mn_2/3_O_2_‐positive electrode material by a simple solid‐state route and high sodium content in this P2‐structure is realized by introducing the Fe element.

The stoichiometry of the as‐synthesized Na_7/9_Cu_2/9_Fe_1/9_Mn_2/3_O_2_ was first determined by an inductively coupled plasma (ICP) analysis to make sure the composition. The measurement result shows that the Na:Cu:Fe:Mn molar ratio is 0.76:0.22:0.11:0.67, which is very close to the expected stoichiometry. We also further confirm the sodium content using the electrochemical method where the electrode was first discharged to 1.8 V; the result is shown in Figure S1 (Supporting Information). The initial discharge capacity is 60 mAh g^−1^, corresponding to 0.24 Na insertion, which indicates that the actual sodium content of the Na_7/9_Cu_2/9_Fe_1/9_Mn_2/3_O_2_ is 0.76. The powder X‐ray diffraction (XRD) pattern and the Rietveld‐refined results of the Na_7/9_Cu_2/9_Fe_1/9_Mn_2/3_O_2_ product are shown in **Figure** **1**a. The diffraction pattern clearly shows a single phase where no crystalline impurities are observed. All the diffraction peaks indicate that the Na_7/9_Cu_2/9_Fe_1/9_Mn_2/3_O_2_ crystallizes in the hexagonal layered structure (P2‐type structure) with the space group P63/mmc, and the peaks are sharp and well defined, demonstrating a good crystallinity. This resulting material is very stable in air and it can keep its original structure even after soaking in water as shown in Figure S2 (Supporting Information). The unit cell parameters obtained from the structural refinement are *a* = 2.9060(6) Å and *c* = 11.1703(1) Å. The schematic illustration of the P2 structure is given in Figure 1b, in which sodium ions are accommodated between MO_2_ slabs consisting of edge‐sharing M (Cu, Fe, Mn) O_6_ octahedral. To minimize electrostatic repulsion between sodium ions, two prismatic sites of Na1 and Na2 that share face or edge with MO_6_ octahedral are simultaneously occupied by Na^+^ ions.

**Figure 1:**
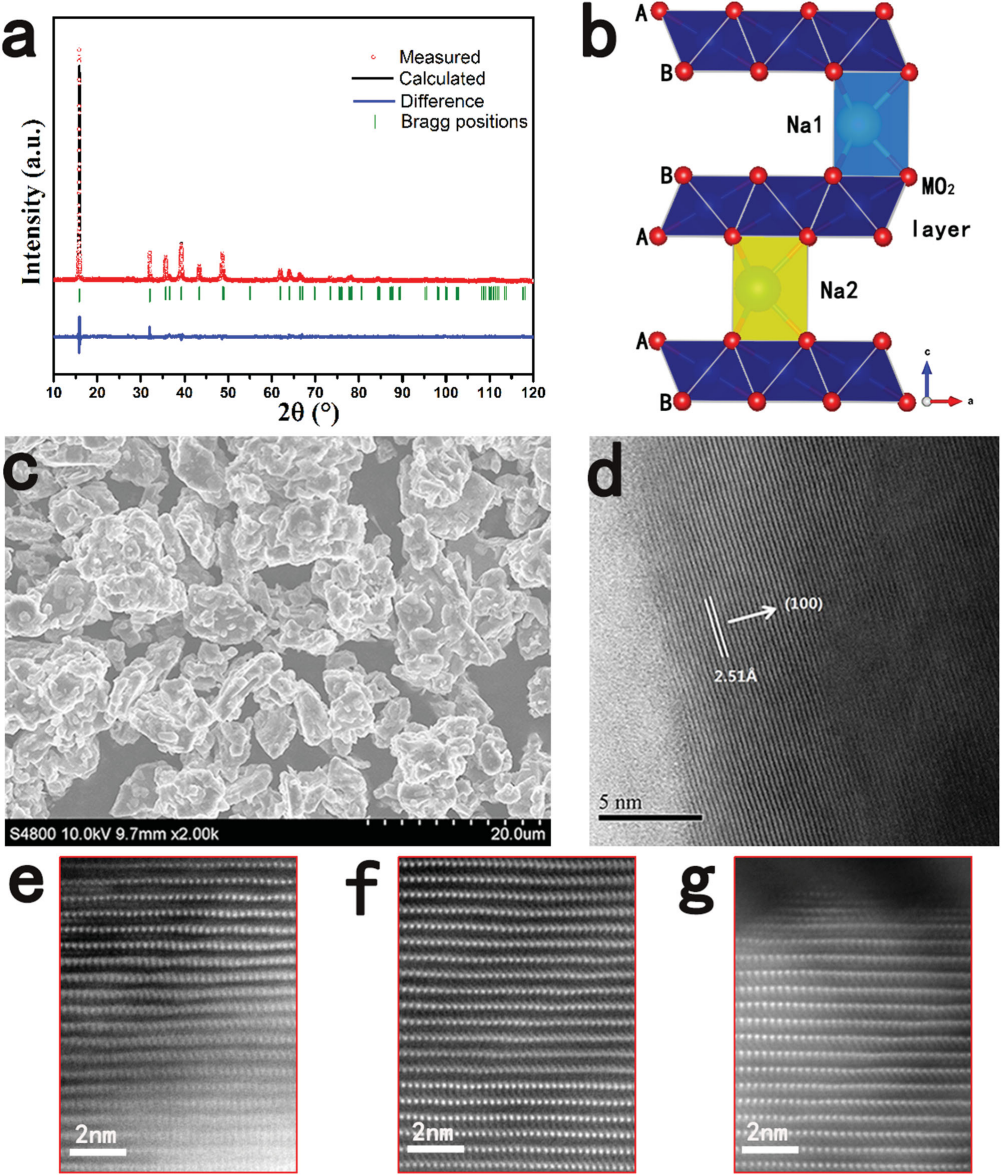
a) Observed and calculated XRD profiles for Na_7/9_Cu_2/9_Fe_1/9_Mn_2/3_O_2_: (red) observed; (black) calculated; (blue) difference plot; (green bars) Bragg reflections. b) Schematic illustration of the P2‐type structure. c) SEM image. d) High‐resolution TEM image of the Na_7/9_Cu_2/9_Fe_1/9_Mn_2/3_O_2_ microflak. HAADF‐STEM image of e) the pristine material, f) the charged state, and g) the discharged state along the [010] direction.

Figure 1c exhibits the SEM image of the morphology of Na_7/9_Cu_2/9_Fe_1/9_Mn_2/3_O_2_. The sample appears as flake‐like particles, which is in good agreement with the layered structure of this material. The particle morphology of Na_7/9_Cu_2/9_Fe_1/9_Mn_2/3_O_2_ is found to be well‐crystallized with 2–10 μm in size even though nonuniform distribution of the particle size is noted. The high‐resolution TEM image in Figure 1d also presents the layered structure of this material clearly, where the interplanar distances between the neighboring lattice fringes can be well defined as 2.51 Å, corresponding to the *d*‐spacing value of the (100) plane of the P2 phase. The bright‐dot contrast in the HAADF STEM image of the pristine material along the [010] direction, as shown in Figure 1e, reveals the transition‐metal (Cu, Fe, and Mn) atom column positions, showing a typical P2‐type structure.

The electrochemical performance of the Na_7/9_Cu_2/9_Fe_1/9_Mn_2/3_O_2_ in sodium half cells was tested by cyclic voltammogram (CV) and galvanostatic charge/discharge cycling. **Figure** **2**a presents the typical CV curves of the Na_7/9_Cu_2/9_Fe_1/9_Mn_2/3_O_2_ electrode versus Na metal between 2.5 and 4.2 V. The main feature in the CV curves is a pair of oxidation/reduction peaks at 4.1 V and 3.9 V, which corresponds to the redox couple of Cu^2+^/Cu^3+^ according to previous reports.^52^ This also indicates that the redox reaction of Cu^2+^/Cu^3+^ contributes most of the capacity in the Na_7/9_Cu_2/9_Fe_1/9_Mn_2/3_O_2_ electrode. The small difference between oxidation potential and reduction potential demonstrates a small polarization and good kinetics. We can also find other oxidation peaks at potentials of 2.8, 3.8, 3.9 V and reduction peaks at potentials of 3.4 V, 3.5 V. The 2.8 V oxidation peak should be contributed by a small quantity of transfer of Mn^3+^ to Mn^4+^; the others correspond to the redox reaction of Fe^3+^/Fe^4+^.^45^, ^54^, ^55^ In the earlier studies of Mn‐based layered compounds, it was found that complicated CV peaks indicated multiple phase transitions as the systems were desodiated.^56^, ^57^ This complexity has been previously attributed to Na^+^/vacancy ordering or phase transition that involves the gliding of oxygen planes.^57^ However, in the case of Na_7/9_Cu_2/9_Fe_1/9_Mn_2/3_O_2_, the CV curves are very simple and peaks are not very obvious, which indicates that the P2‐Na_7/9_Cu_2/9_Fe_1/9_Mn_2/3_O_2_ structure is stable in the process of electrochemical reaction. We also tested the Na^+^ ion diffusion coefficient of this material by CV (as shown in Figure S3 in the Supporting Information); the estimated diffusion coefficient is ≈2.7 × 10^−11^ cm^2^ s^−1^.

**Figure 2:**
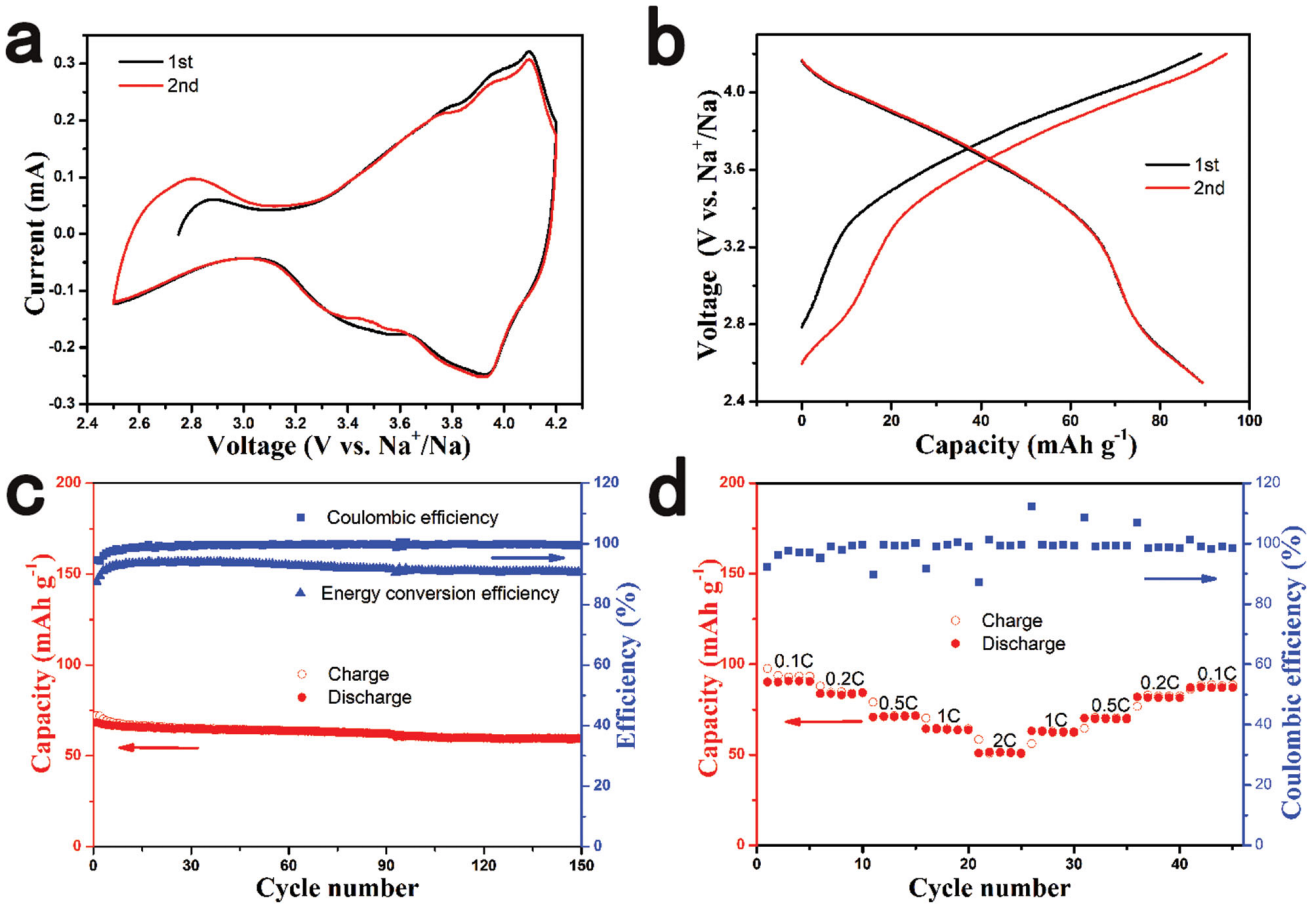
The electrochemical performance of the Na_7/9_Cu_2/9_Fe_1/9_Mn_2/3_O_2_ electrode. a) Cyclic voltammetry (CV) curves at a scanning rate of 0.2 mV s^−1^. b) Charge/discharge curves at 0.1 C rate. c) Cycling performance at 1 C rate. d) Rate performance at various current rates from 0.1 C to 2 C.

Figure 2b shows the first and second charge/discharge profiles of the Na_7/9_Cu_2/9_Fe_1/9_Mn_2/3_O_2_ electrode at 0.1C rate (10 mA g^−1^). The initial discharge capacity is 89 mAh g^−1^, which corresponds to 0.356 e^−^ transfer. The performance can be comparable with nickel‐based oxides; for example, the Na_2/3_Ni_1/3_Mn_2/3_O_2_ delivers 88 mAh g^−1^ in a voltage range of 2.5–4.2 V with a lower storage voltage as shown in Figure S5 (Supporting Information). Figure S5 (Supporting Information) also compares the electrochemical performance of the Na_7/9_Cu_2/9_Fe_1/9_Mn_2/3_O_2_ and the Na_2/3_Cu_2/9_Fe_1/9_Mn_2/3_O_2_ (XRD pattern is shown in Figure S4 in the Supporting Information); we can find that high sodium content is beneficial not only for enhancing the capacity but also for improving the storage voltage. The profile can be divided into two sloping regions, which indicates that a solid solution reaction instead of phase transition occurs upon Na extraction and insertion. In addition, the sloping curves also indicate the disordered arrangement of Cu, Fe, and Mn, and there is no Na^+^/vacancy ordering arrangement, which is in sharp contrast to Na_2/3_Ni_1/3_Mn_2/3_O_2_. The discharge and charge curves are highly symmetrical in the second cycle with a high energy conversion efficiency of 91%, presenting a small polarization of the Na_7/9_Cu_2/9_Fe_1/9_Mn_2/3_O_2_ electrode. The average potential for the charge and discharge processeses up to 3.6 V, which is a high voltage for layered transition‐metal oxides.

Figure 2c exhibits the cycling performance of Na_7/9_Cu_2/9_Fe_1/9_Mn_2/3_O_2_ at a constant current rate of 1C during the initial 150 cycles. The Na_7/9_Cu_2/9_Fe_1/9_Mn_2/3_O_2_ electrode displays excellent cycling performance with only a slow decay over the first several cycles. The discharge capacity after the 150th cycle remains 59.5 mAh g^−1^, which is 87% of the initial discharge capacity. The initial energy conversion efficiency is 87%, and it can remain above 90% after the third cycle. The Coulombic efficiency during the cycles reaches above 98% after ten cycles.

The rate performance of the Na_7/9_Cu_2/9_Fe_1/9_Mn_2/3_O_2_ electrode was also evaluated as shown in Figure 2d. The cells were charged and discharged at various rates from 0.1C to 2C for five cycles. The reversible capacities are 90, 83, 71, 64, and 51 mAh g^−1^ at constant current rates of 0.1C, 0.2C, 0.5C, 1C, and 2C, respectively, suggesting a relatively good rate capability. The capacity can return to the previous value when the current rate is reduced, suggesting a good reversibility of the Na_7/9_Cu_2/9_Fe_1/9_Mn_2/3_O_2_ under a wide current range. We believe that the rate performance can be further improved by doping other transition metals or decreasing particle size.

In order to understand the structure evolution of the Na_7/9_Cu_2/9_Fe_1/9_Mn_2/3_O_2_ electrode during the first charge and discharge process, we performed the electrochemical in situ XRD experiment and the results are presented in **Figure** **3**. The peaks marked with black asterisk belong to Al foil that is used as X‐ray window. No new peaks beyond a P2 structure were observed during sodium extraction and insertion, but only some gradual shift of peak position. That suggests a solid–solution reaction mechanism during sodium extraction/insertion, which is coincident with CV and electrochemical charge/discharge curves. The evolution of the *a* and *c* lattice parameters during the first charge/discharge is shown in Figure S6 (Supporting Information); the unit‐cell volume change before and after sodium extraction is only 1.32%. In addition, the peak intensity does not fade in the process of electrochemical reaction, which indicates that the structure of P2‐Na_7/9_Cu_2/9_Fe_1/9_Mn_2/3_O_2_ is electrochemically stable even though the cell is charged to a high cutoff voltage of 4.2 V. In contrast, O2‐type stacking faults are introduced into the nickel‐based P2 phase electrode material when charging cutoff voltage is above 4.2 V. The structural stability of P2‐Na_7/9_Cu_2/9_Fe_1/9_Mn_2/3_O_2_ should be due to the high sodium content, which makes a large amount of residual Na^+^ (≈0.4) can be maintained in the layer structure. (Note that if the sodium content is below 1/3, then a phase transition from P2 to O2 occurs.) During the first charge, the (002) and (004) peaks shift to a lower angle, while the (100) peak shifts to a higher angle, indicating that the *c*‐axis is expanding and the *a‐*, *b*‐axis are contracting when sodium is extracted. When discharging to 2.5 V, these peaks can return to their initial states, which shows the highly reversible change of lattice parameter and structural stability.

**Figure 3:**
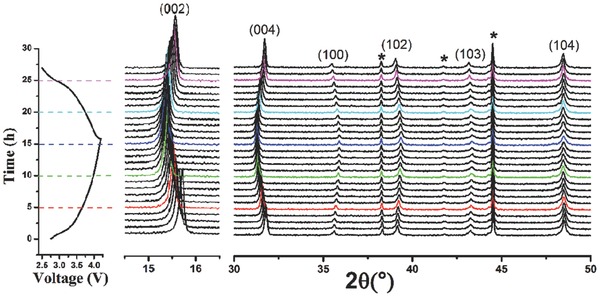
In situ XRD patterns collected during the first charge/discharge of the Na_7/9_Cu_2/9_Fe_1/9_Mn_2/3_O_2_ electrode between 2.5 V and 4.2 V at C/15 rate. Black asterisks represent peaks of Al window.

The structural stability can also be proved by the HAADF STEM images as shown in Figure 1f,g. The charged state and the discharged state have the same HAADF STEM images with the pristine material except for the difference of the interplanar distances, which exhibits that the Na_7/9_Cu_2/9_Fe_1/9_Mn_2/3_O_2_ could keep the P2‐type structure during Na extraction and insertion. In short, by raising the sodium content, we successfully stabilize the P2‐type layered structure even though it is charged to a high voltage of 4.2 V.

To investigate the changes in the oxidation states of Cu, Fe, and Mn during the charge process, XPS and EELS experiments were performed for the Na_7/9_Cu_2/9_Fe_1/9_Mn_2/3_O_2_ electrodes. **Figure** **4**a–c displays the comparison of the binding energies of Cu 2p, Fe 2p, and Mn 2p from the electrodes before and after charged to 4.2 V. In the case of the pristine sample, the binding energies of Cu 2p_3/2_, Fe 2p_3/2_, and Mn 2p_3/2_ are 933.2, 711.3, and 641.8 eV, respectively, corresponding to the valences of Mn^3+^ and Mn^4+^, while the valence states of Cu and Fe are divalent and trivalent in accordance with the precursor materials. When fully charged to 4.2 V, the binding energies of Cu, Fe, and Mn move to higher energy values with the shifts of 0.4, 0.35, and 0.3 eV, respectively, which confirm the oxidations of Cu^2+^ to Cu^3+^, Fe^3+^ to Fe^4+^, and Mn^3+^ to Mn^4+^ upon Na extraction.^55^ These changes indicate that Cu, Fe, and Mn all take part in the charge compensation, which are further supported by the EELS results as shown in Figure 4d–f. The EELS spectra of Cu L_2,3_, Fe L_2,3_, and Mn L_2,3_ all shift to higher energy values with 1.5, 1.4, and 1.7 eV shifts upon charged to 4.2 V.^58^, ^59^, ^60^ This is the first time to prove the Cu^2+^/Cu^3+^ redox couple in P2 phase oxides.

**Figure 4:**
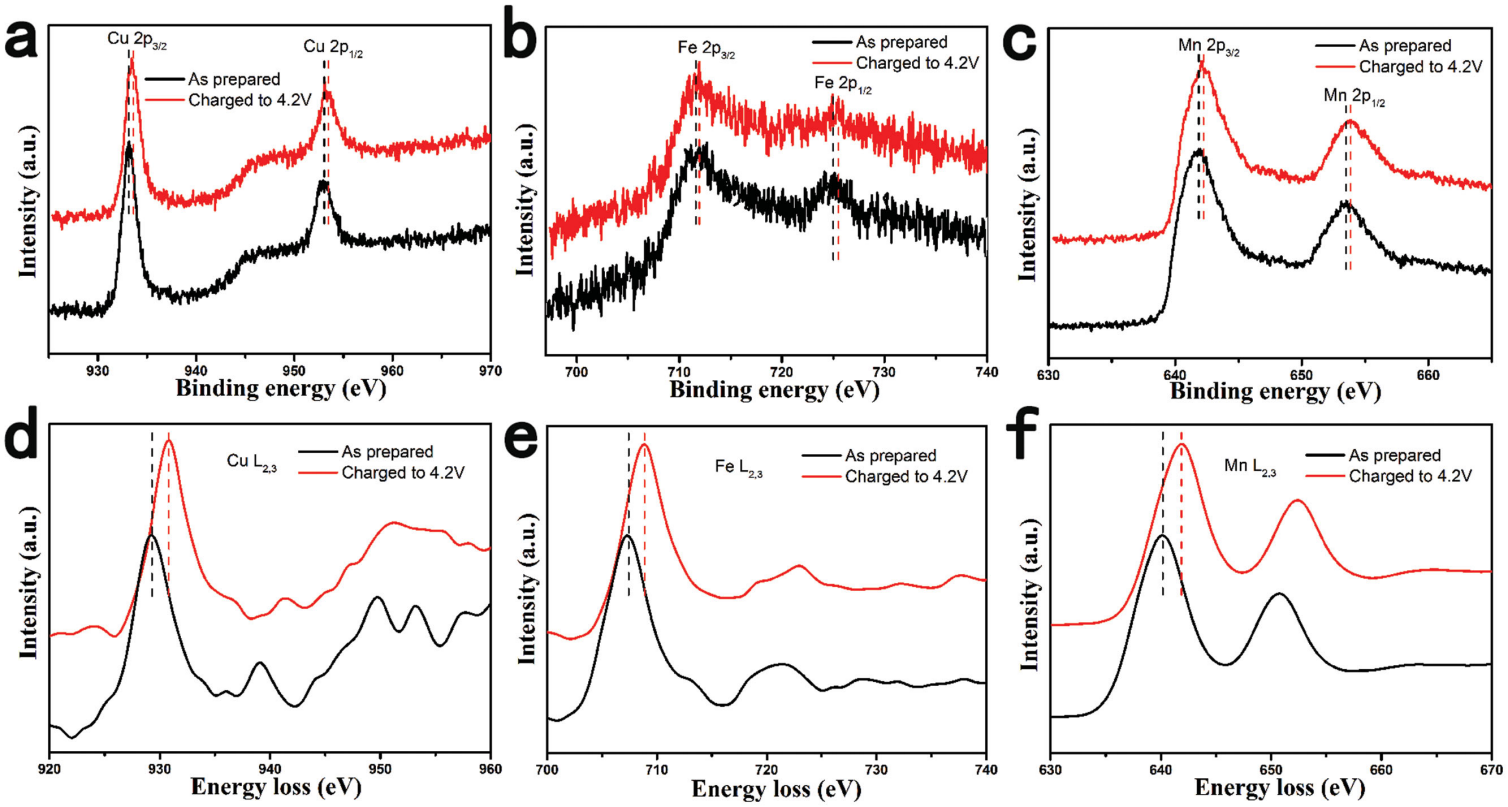
XPS spectra of a) Cu 2p, b) Fe 2p, c) Mn 2p and EELS spectra of d) Cu L_2,3_, e) Fe L_2,3_, f) Mn L_2,3_ edges of the Na_7/9_Cu_2/9_Fe_1/9_Mn_2/3_O_2_ electrode before and after charged to 4.2 V.

To demonstrate the application prospects of the sample, we fabricated a sodium‐ion full battery with the Na_7/9_Cu_2/9_Fe_1/9_Mn_2/3_O_2_‐positive electrode and the hard carbon negative electrode. The charge and discharge curves of the hard carbon negative electrode at 0.1C are shown in Figure S7 (Supporting Information). The full cell was cycled at 0.2C, and the preliminary results of electrochemical measurement are displayed in **Figure** **5**. The Na_7/9_Cu_2/9_Fe_1/9_Mn_2/3_O_2_/hard carbon full cell delivers a high capacity of 313 mAh g^−1^ (based on the negative electrode) and a high initial Coulombic efficiency of 79%, respectively. The average operation voltage is around 3.5 V. The energy density of this system is calculated to be 195 Wh kg^−1^ with a high energy conversion efficiency of above 85% after the first cycle, indicating a small polarization. The Na_7/9_Cu_2/9_Fe_1/9_Mn_2/3_O_2_/Hard carbon full cell also exhibits superior cycle performance as shown in Figure 5b with a capacity retention of 89% after 50 cycles. These desired promising properties are believed to make the system closer to practical application with high energy density.

**Figure 5:**
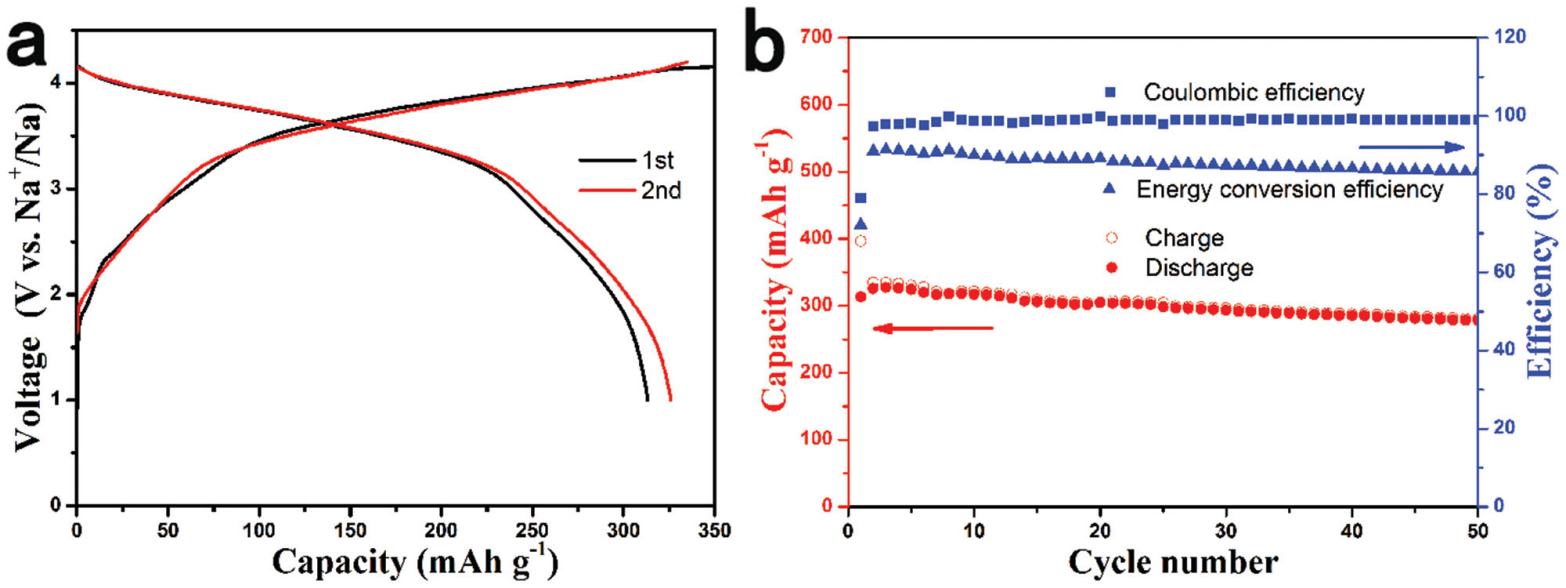
The Na storage performance of a Na_7/9_Cu_2/9_Fe_1/9_Mn_2/3_O_2_/Hard carbon full cell cycled at 0.2C rate. a) Charge/discharge curves for the first and second cycles and b) cycling performance.

In summary, we designed and prepared a novel air‐stable P2‐Na_7/9_Cu_2/9_Fe_1/9_Mn_2/3_O_2_ with high sodium content and investigated its electrochemical performance as a positive electrode material for rechargeable sodium‐ion batteries. This material exhibits a reversible capacity of 89 mAh g^−1^ at 0.1C rate. The attractive performance is the long cycling stability as demonstrated by the capacity retention of 85% after 150 cycles at 1C rate without phase transformation. The Na_7/9_Cu_2/9_Fe_1/9_Mn_2/3_O_2_ electrode material also displays a high initial Coulombic efficiency, a high average operating voltage of 3.6 V, and a small polarization, and it does not contain toxic and expensive transition metals. The results of XPS and EELS experiments prove the reversible Cu^2+^/Cu^3+^ redox couple in P2 phase oxides for the first time. When coupled with hard carbon negative electrode, promising application prospects were demonstrated with a high reversible capacity of 313 mAh g^−1^, a high initial Coulombic efficiency of 79%, and a high energy density of 195 Wh kg^−1^ at 0.2C rate. These outstanding performances of the air‐stable P2‐Na_7/9_Cu_2/9_Fe_1/9_Mn_2/3_O_2_ would pave the way for practical application in large‐scale electrical energy storage with only environmentally friendly and low‐cost elements.

## Experimental Section

*Materials Synthesis and Characterizations*: Na_7/9_Cu_2/9_Fe_1/9_Mn_2/3_O_2_ was synthesized by a conventional solid‐state reaction. The precursor materials of Na_2_CO_3_, CuO, Fe_2_O_3_, and Mn_2_O_3_ were mixed and ground uniformly in stoichiometric proportion, and the resulting material was pressed into pellets under pressure of 20 MPa. Then, the pellets were heated at 900 °C for 15 h in an alumina crucible. The actual ratios of Na:Cu:Fe:Mn were verified by inductively coupled plasma‐atomic emission spectroscopy (ICP‐AES, Shimadzu, ICPS‐8100). The morphology of the sample was investigated with a scanning electron microscope (Hitachi S‐4800). High‐resolution transmission electron microscope (HRTEM) and electron energy loss spectroscopy (EELS) patterns were recorded on a FEI Tecnai F20 transmission electron microscope. STEM was performed using a JEOL 2100F (JEOL, Tokyo, Japan) transmission electron microscope operated at 200 keV. The crystalline structures were characterized by X‐ray diffraction, using a Bruker D8 Advance Diffractometer in a transition mode using Cu Kα radiation (1.5405 Å). The crystal structure was refined using the Rietveld method as implemented in the TOPAS software package. For in situ XRD experiment during electrochemical cycling, a special cell was used with an aluminum window for X‐ray penetration. The in situ cell was charged and discharged at a current rate of C/15. The X‐ray photoelectron spectroscopy (XPS) spectra were recorded with a spectrometer having Mg/Al Kα radiation (ESCALAB 250 Xi, ThermoFisher). All binding energies reported were corrected using the signal of the carbon at 284.8 eV as an internal standard.

*Electrochemical Measurements*: The working electrodes were prepared by spreading the slurry of the active materials (80 wt%), acetylene black (10 wt%), and the polyvinylidene fluoride (PVdF, 10 wt%) on Al foil, and then dried at 120 °C under vacuum for 10 h to remove the solvent. All the electrochemical tests were conducted in coin cells (CR2032). The coin cells were assembled with sodium metal as a counter electrode, 0.8 m NaPF_6_ in PC as an electrolyte, and glass fiber as a separator in an argon‐filled glove box. The charge and discharge tests were carried out on a Land BT2000 battery test system (Wuhan, China) in a voltage range of 2.5–4.2 V under room temperature. The “energy conversion efficiency” was calculated by dividing the discharge energy by charge energy (discharge energy/charge energy). Cyclic voltammetry (CV) was measured using Autolab PGSTAT302N (Metrohm, Switzerland). A sodium‐ion full cell was constructed using Na_7/9_Cu_2/9_Fe_1/9_Mn_2/3_O_2_ as the positive electrode and hard carbon as the negative electrode in a CR2032 coin‐type cell. The hard carbon was prepared as described in our previous work[[qv: 10b]] by pyrolysis of sugar at 1600 °C. The hard carbon electrode was prepared by mixing the active material and PVdF in a weight ratio of 95:5. The weight ratio of the two electrodes (negative/positive) was 1:4.434. Note that hard carbon exhibits a lower initial Coulombic efficiency in 0.8 m NaPF_6_–PC electrolyte (as shown in Figure S8 in the Supporting Information), thereby in the full cell study, we chose the 1 m NaClO_4_ in EC:DEC (1:1) as an electrolyte. The full cell was charged and discharged in the voltage range of 1–4.2 V at a current rate of 0.2C. The specific capacity of full battery is calculated based on the mass of the negative electrode material.

## Supporting information

As a service to our authors and readers, this journal provides supporting information supplied by the authors. Such materials are peer reviewed and may be re‐organized for online delivery, but are not copy‐edited or typeset. Technical support issues arising from supporting information (other than missing files) should be addressed to the authors.

Supplementary
