# Supplementary material for: Air‐Stable Copper‐Based P2‐Na7/9Cu2/9Fe1/9Mn2/3O2 as a New Positive Electrode Material for Sodium‐Ion Batteries
Source: Adv Sci (Weinh). 2015 May 4;2(6):1500031. doi: 10.1002/advs.201500031 (PMC5115408; doi:10.1002/advs.201500031)
Supplement: Supplementary file 1 — Supplementary [file ADVS-2-0b-s001.pdf]

Copyright WILEY-VCH Verlag GmbH & Co. KGaA, 69469 Weinheim, Germany, 2015.

ADVANCED SCIENCE
Open Access

# Supporting Information

for *Adv. Sci.*, DOI: 10.1002/advs.201500031

# Air-Stable Copper-Based P2-$Na_{7/9}Cu_{2/9}Fe_{1/9}Mn_{2/3}O_2$ as a New Positive Electrode Material for Sodium-Ion Batteries

*Yunming Li, Zhenzhong Yang, Shuyin Xu, Linqin Mu, Lin Gu,* Yong-Sheng Hu,* Hong Li, and Liquan Chen*

Supporting Information

**Air-stable copper-based P2-$Na_{7/9}Cu_{2/9}Fe_{1/9}Mn_{2/3}O_2$ as a new positive electrode material for sodium-ion batteries**

*By Yunming Li, Zhenzhong Yang, Shuyin Xu, Linqin Mu, Lin Gu*, Yong-Sheng Hu*, Hong Li, Liquan Chen*

Prof. Y.-S. Hu, Y. M. Li, S. Y. Xu, L. Q. Mu, Prof. H. Li, Prof. L. Q. Chen
Key Laboratory for Renewable Energy, Beijing Key Laboratory for New Energy Materials and Devices, Beijing National Laboratory for Condensed Matter Physics, Institute of Physics, Chinese Academy of Sciences, Beijing100190, China
E-mail: yshu@aphy.iphy.ac.cn

Prof. L. Gu, Z. Z. Yang
Laboratory for Advanced Materials & Electron Microscopy, Institute of Physics, Chinese Academy of Sciences, Beijing 100190, China
E-mail: l.gu@iphy.ac.cn

**Author contributions**

Y.-S.H. conceived and designed this work; Y.M.L. performed all the synthesis and electrochemical experiments; L.G. and Z.Z.Y. performed STEM and EELS experiments; Y.M.L. and Y.-S.H. wrote the paper; all the authors participated in analysis of the experimental data and discussions of the results as well as preparing the paper.

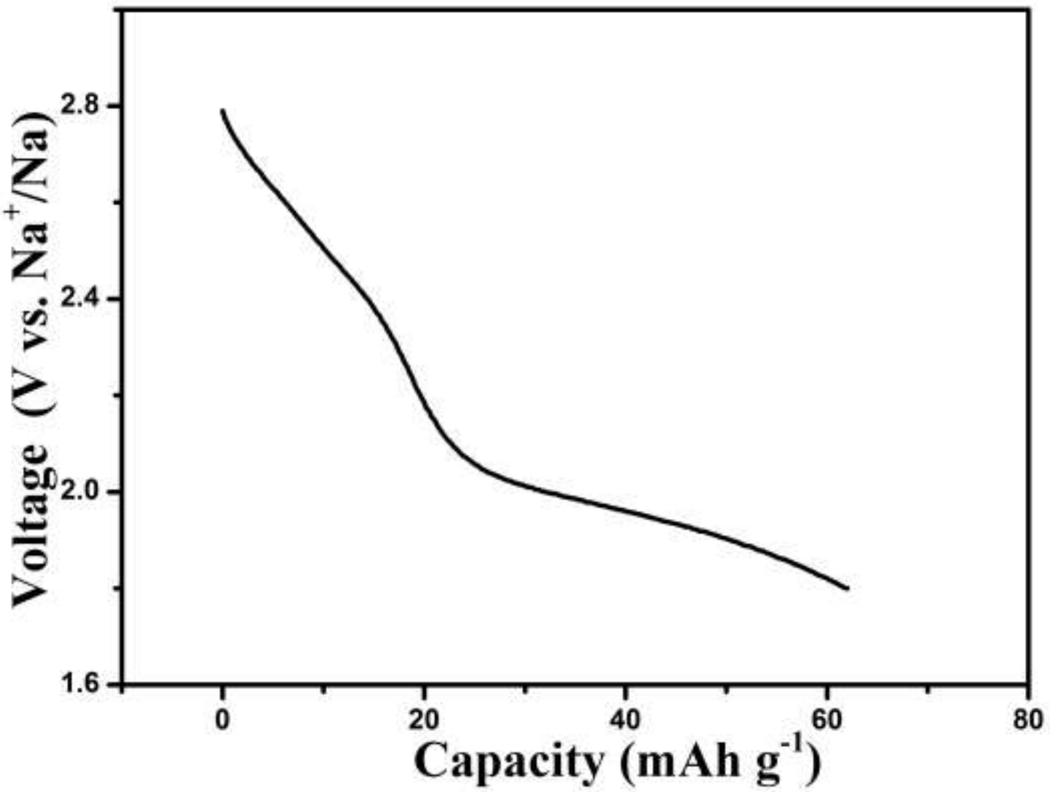


**Figure S1.** Discharge curve for the first cycle of the P2-$Na_{7/9}Cu_{2/9}Fe_{1/9}Mn_{2/3}O_2$ electrode under the condition of discharging first.

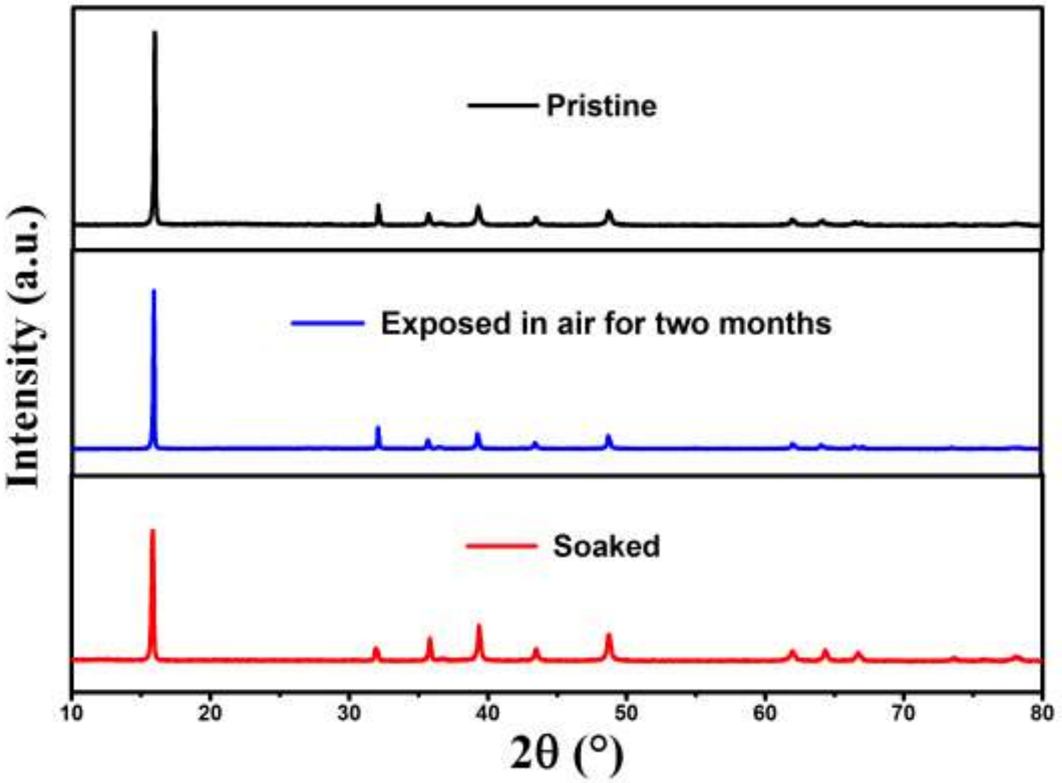


**Figure S2.** XRD patterns of the pristine P2-$Na_{7/9}Cu_{2/9}Fe_{1/9}Mn_{2/3}O_2$ sample, exposed in air for two months and after soaking into water.

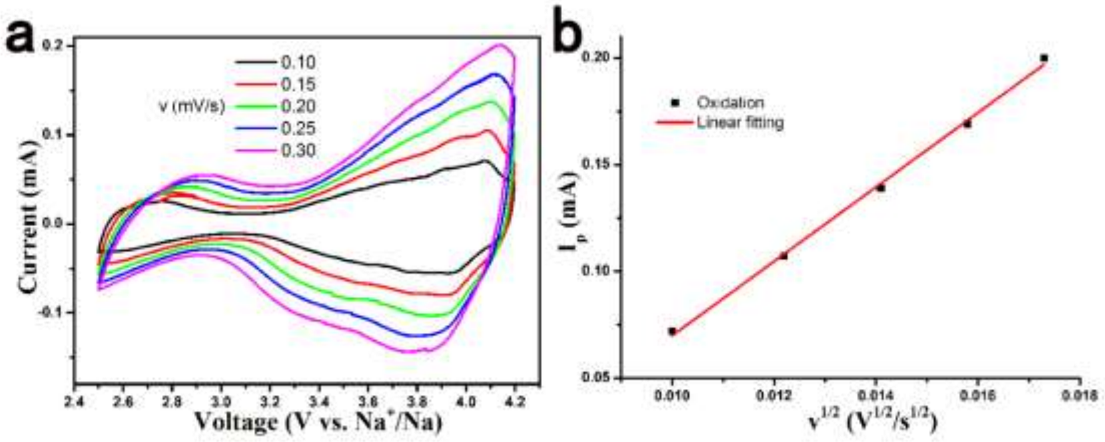


**Figure S3.** $Na^+$ ion diffusion kinetics in P2-$Na_{7/9}Cu_{2/9}Fe_{1/9}Mn_{2/3}O_2$. (a) Cyclic voltammograms of the P2-$Na_{7/9}Cu_{2/9}Fe_{1/9}Mn_{2/3}O_2$ electrodes at various scan rates, and (b) Relationship between peak current $I_p$ and square root of scan rate $v^{1/2}$. The $Na^+$ ion diffusion coefficient was calculated to be ~$2.7\times10^{-11}$ $cm^2$ $s^{-1}$. (Note that The $Na^+$ ion apparent diffusion coefficient was calculated according to the Randles-Sevcik equation:

$$I_p = 0.4463\mathrm{nFAC}(\mathrm{nFD}v/\mathrm{RT})^{1/2}$$

$I_p$ is peak current, n is the number of moles of electrons transferred in the reaction, F is Faraday constant, A is the area of the electrode, C is the extracted $Na^+$ ion concentration (in $mol/cm^3$) in $Na_{7/9}Cu_{2/9}Fe_{1/9}Mn_{2/3}O_2$, D is the apparent diffusion coefficient, and $v$ is the scan rate.)

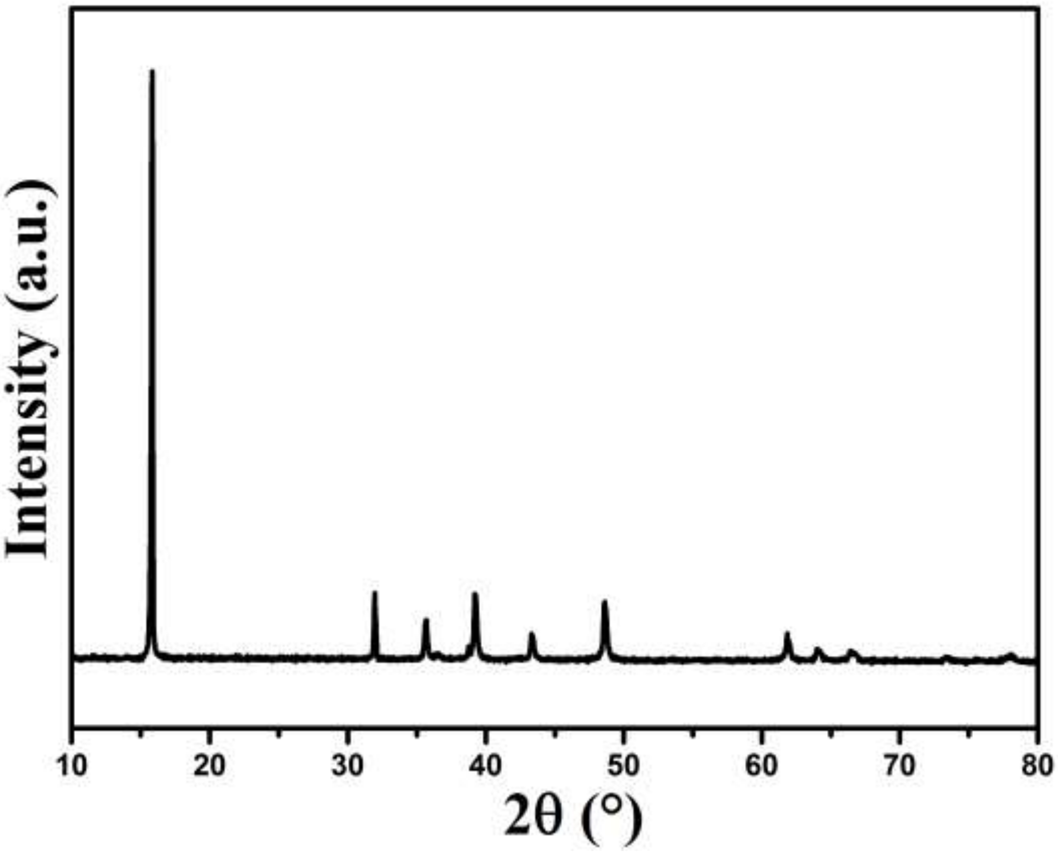


**Figure S4.** XRD pattern of the P2-$Na_{2/3}Cu_{2/9}Fe_{1/9}Mn_{2/3}O_2$ sample.

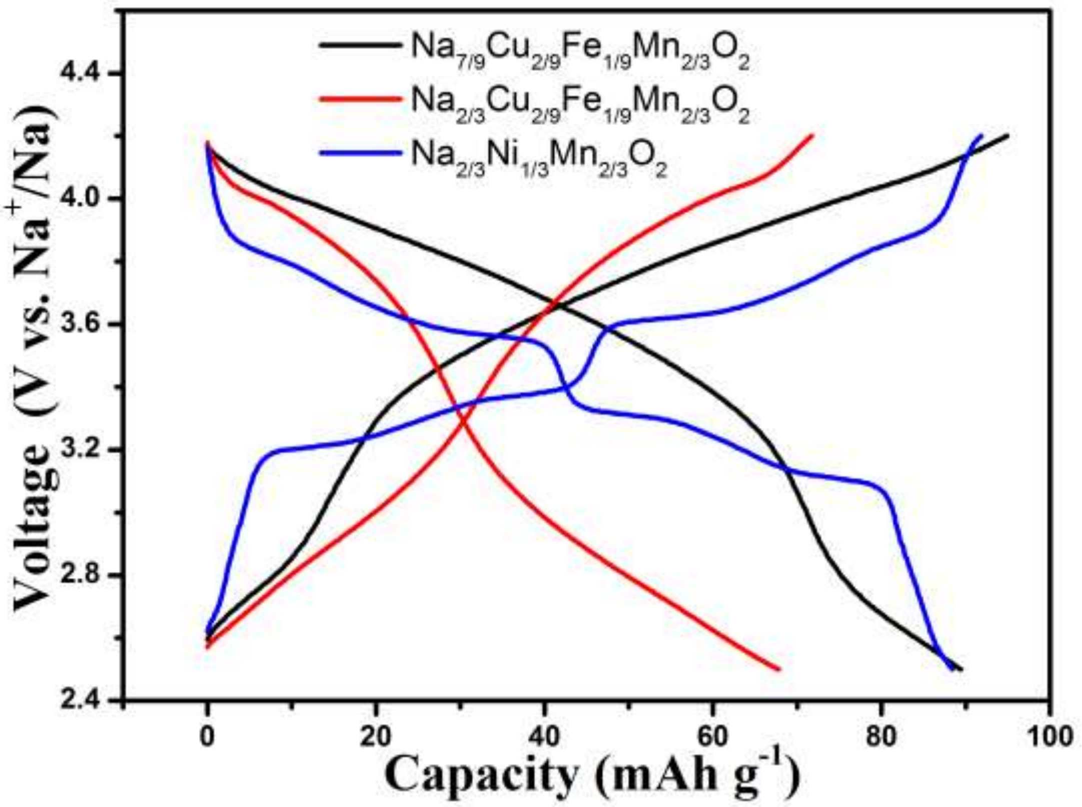


**Figure S5.** Charge-discharge curves for the second cycle of $Na_{7/9}Cu_{2/9}Fe_{1/9}Mn_{2/3}O_2$, $Na_{2/3}Cu_{2/9}Fe_{1/9}Mn_{2/3}O_2$ and $Na_{2/3}Ni_{1/3}Mn_{2/3}O_2$.

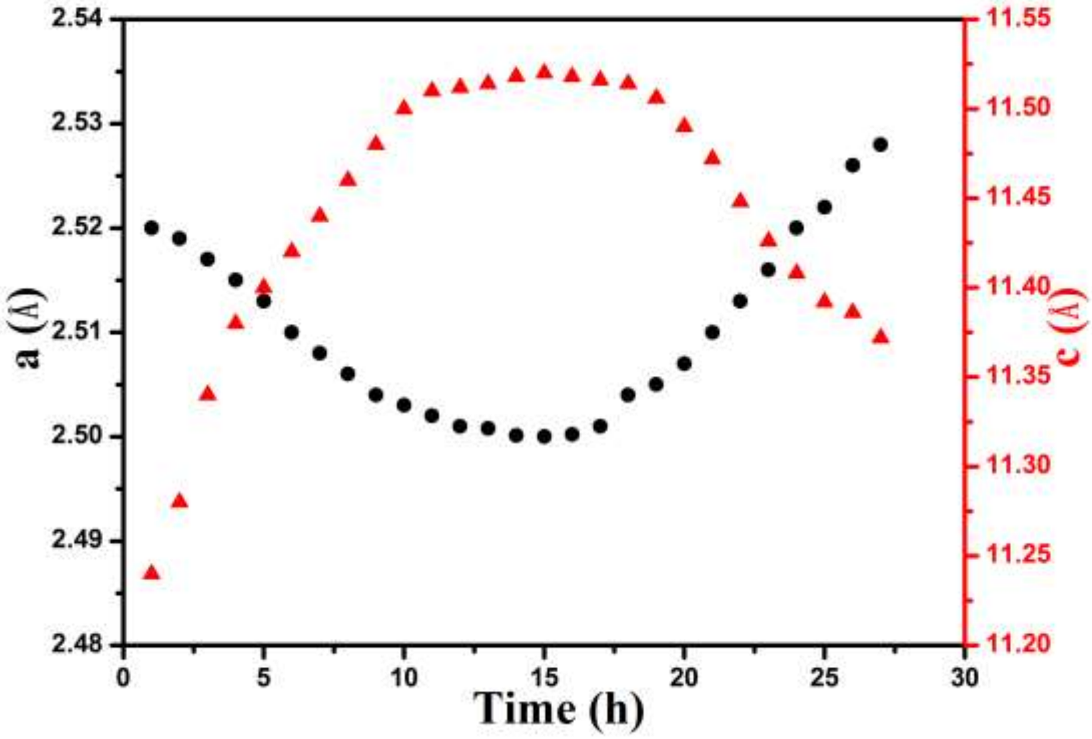


**Figure S6.** The evolution of the *a* and *c* lattice parameters during the first charge/discharge process.

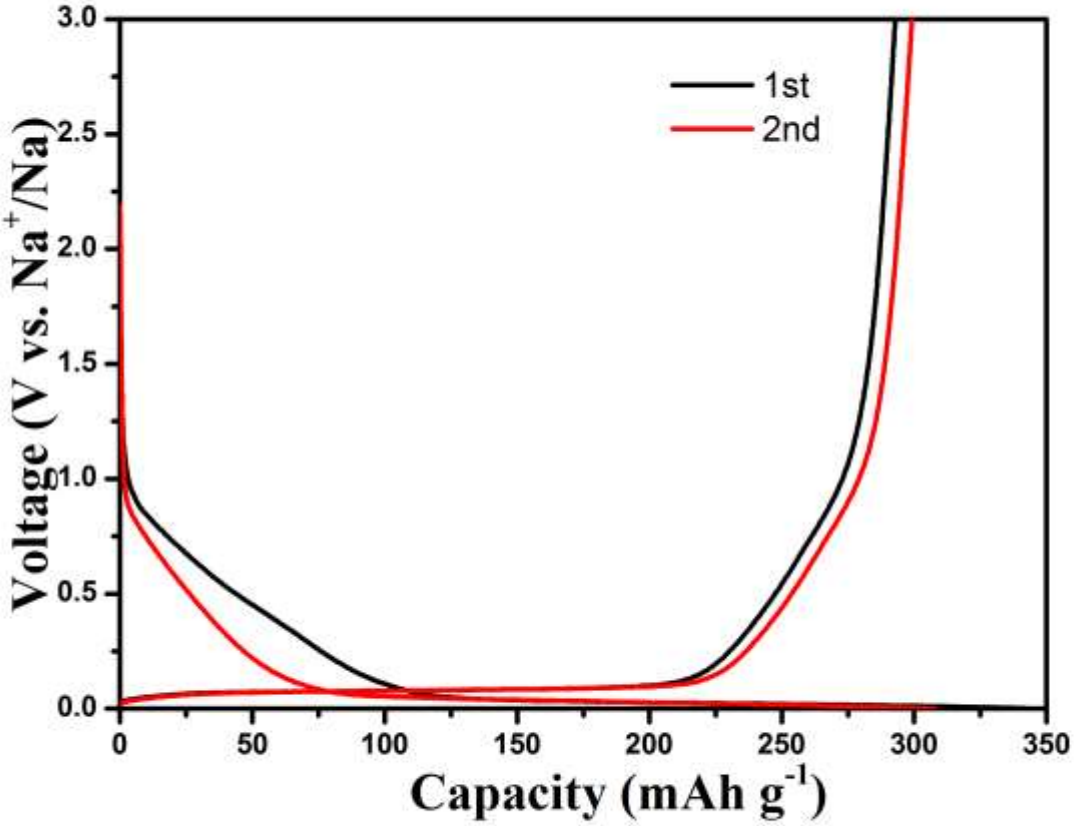


**Figure S7.** Charge-discharge curves for the 1st, 2nd cycles of the hard carbon negative electrode at 0.1C rate in the 0.8 M $NaPF_6$ - PC electrolyte.

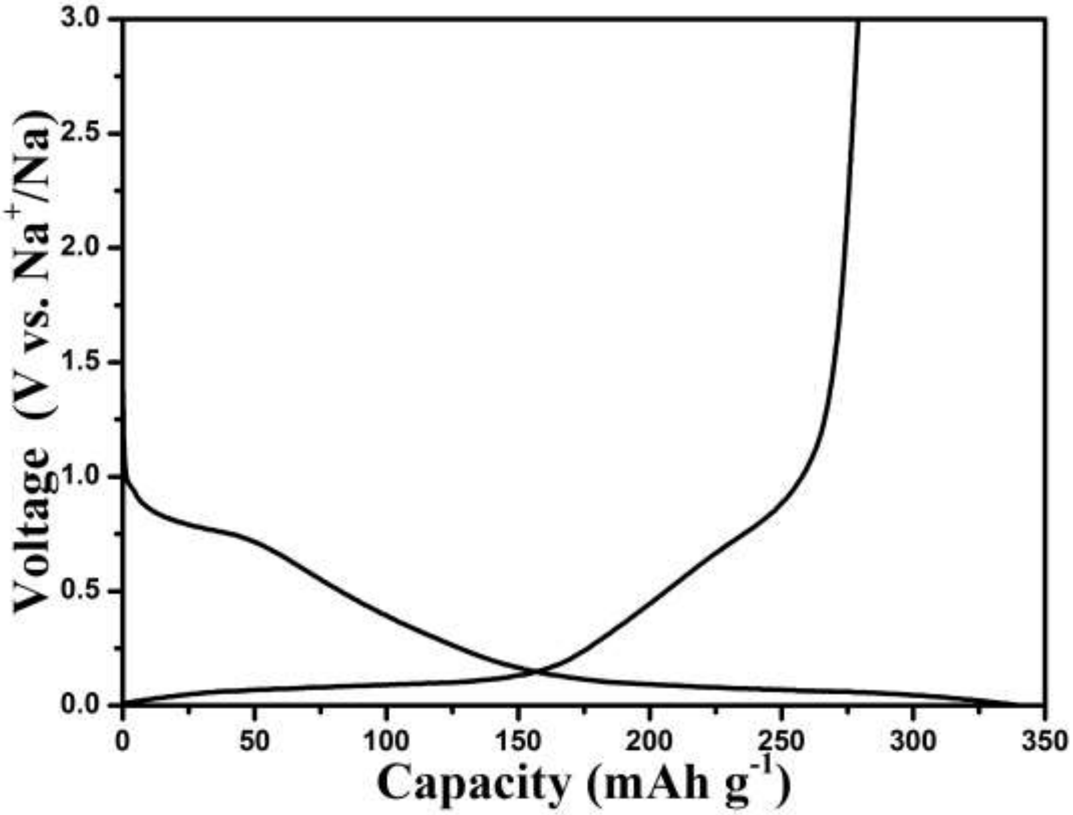


**Figure S8.** Charge-discharge curves for the 1st cycle of the hard carbon negative electrode at 0.1C rate in the 1 M $NaClO_4$ - EC:DEC (1:1) electrolyte.
